# MDM2 Contributes to High Glucose-Induced Glomerular Mesangial Cell Proliferation and Extracellular Matrix Accumulation via Notch1

**DOI:** 10.1038/s41598-017-10927-5

**Published:** 2017-09-04

**Authors:** Chun-Tao Lei, Hui Tang, Chen Ye, Chao-Qun You, Jiao Zhang, Chun-Yun Zhang, Wei Xiong, Hua Su, Chun Zhang

**Affiliations:** Department of Nephrology, Union Hospital, Tongji Medical College, Huazhong University of Science and Technology, Wuhan, 430022 China

## Abstract

Murine double minute 2 (MDM2) is an E3-ubiquitin ligase critical for various biological functions. Previous data have revealed an indispensable role of MDM2 in kidney homeostasis. However, its role in glomerular mesangial cell (GMC) proliferation and extracellular matrix (ECM) accumulation during hyperglycemia condition remains unclear. In our present study, we found that MDM2 protein level was significantly upregulated in high glucose-treated GMCs, while knocking down MDM2 by siRNA could attenuate high glucose-induced ECM accumulation and GMCs proliferation. Unexpectedly, Nutlin-3a, a MDM2-p53 interaction blocker, had no benefit in protecting diabetic mice from renal impairment *in vivo* and in alleviating high glucose-induced ECM accumulation *in vitro*. Intriguingly, we found that Notch1 signaling activation was obviously attenuated by MDM2 depletion in GMCs with high glucose exposure. However, Numb, a substrate of MDM2 which suppresses Notch1 signaling, was found not to be involved in the MDM2 and Notch1 association. Moreover, our findings demonstrated that MDM2 interacted with Notch1 intracellular domain (NICD1) independent of Numb and regulated the ubiquitination status of NICD1. Collectively, our data propose a pivotal role of MDM2 in high glucose-induced GMC proliferation and ECM accumulation, via modulating the activation of Notch1 signaling pathway in an ubiquitination-dependent way.

## Introduction

Diabetic kidney disease (DKD) is one of the most serious microvascular complications of both type 1 and type 2 diabetic mellitus, resulting in end stage renal disease (ESRD)^1, 2^. Unfortunately, there has been no satisfying intervention strategy for DKD until now, especially in its early stage. Glomerular mesangial cell (GMC) proliferation and excessive extracellular matrix (ECM) deposition in mesangium are hallmark pathological features in the onset and progression of DKD, which lead to glomerulosclerosis and deterioration of renal function^3, 4^. However, the mechanisms mediating GMC proliferation and ECM accumulation under hyperglycemia condition have not been fully clarified.

Murine double minute-2 (MDM2) is an important E3 ubiquitin ligase which promotes the nuclear exportation, ubiquitination and proteasomal degradation process of p53^5–7^. In this regard, MDM2 is considered as an onco-protein by accelerating tumor growth and promoting cancer progression in a p53-dependent manner^8–10^; hence, small molecule inhibitors targeting MDM2-p53 interaction, such as Nutlin-3a, have been developed for the treatment of various types of cancer and are under clinical trial presently^11–13^. Furthermore, MDM2 also correlates to various physiological and pathological processes independent of p53. For example, MDM2 can exert its oncogenic profile via activating Akt signaling^14^, and facilitate sterile inflammation through NF-κB pathway^15^.

MDM2 is widely presented in kidney resident cells including glomerular endothelial cells (GECs), podocytes, parietal epithelial cells (PECs) and tubular epithelial cells (TECs)^16, 17^. Physiologically, MDM2 is indispensable for resting podocytes and TECs in maintaining cell homeostasis and survival by protecting them from p53-related cell death. Knocking down MDM2 results in functional and structural abnormalities of podocytes and TECs^18, 19^. Meanwhile, MDM2 dysregulation is also implicated in the pathological events of various kidney diseases. For example, a German group has demonstrated that MDM2 plays a dual role in post-ischemic acute tubular injury, as it promotes renal sterile inflammation in a NF-κB-dependent manner and drives tubular cell regeneration by blocking p53-mediated cell cycle arrest and apoptosis^16^. Additionally, we recently found MDM2 upregulation is implicated in renal fibroblast activation, leading to tubulointerstitial fibrosis (TIF)^20^. In glomerular injury disease, MDM2 promotes PEC proliferation and intraglomerular inflammation during anti-GBM serum induced-crescentic glomerulonephritis^17^, and also leads to podocyte loss by facilitating cell entering mitosis (i.e., mitotic catastrophe) in adriamycin nephropathy^21^. However, whether MDM2 contributes to the pathogenesis of DKD is rarely reported, thus we are interested in exploring its role in GMC proliferation and ECM accumulation, which are the critical components of DKD.

In our present study, we tested the hypothesis that MDM2 could drive GMC proliferation and ECM accumulation under hyperglycemia condition, and then explored its related downstream signaling pathways. Our findings demonstrate that MDM2 plays a crucial role in high glucose-induced GMC proliferation and ECM accumulation, through modulating the activation status of Notch1 signaling pathway in an ubiquitination-dependent manner, independent of p53.

## Results

### High glucose stimulates cell proliferation and ECM accumulation and induces the upregulation of MDM2 level in GMCs

**Figure 1:**
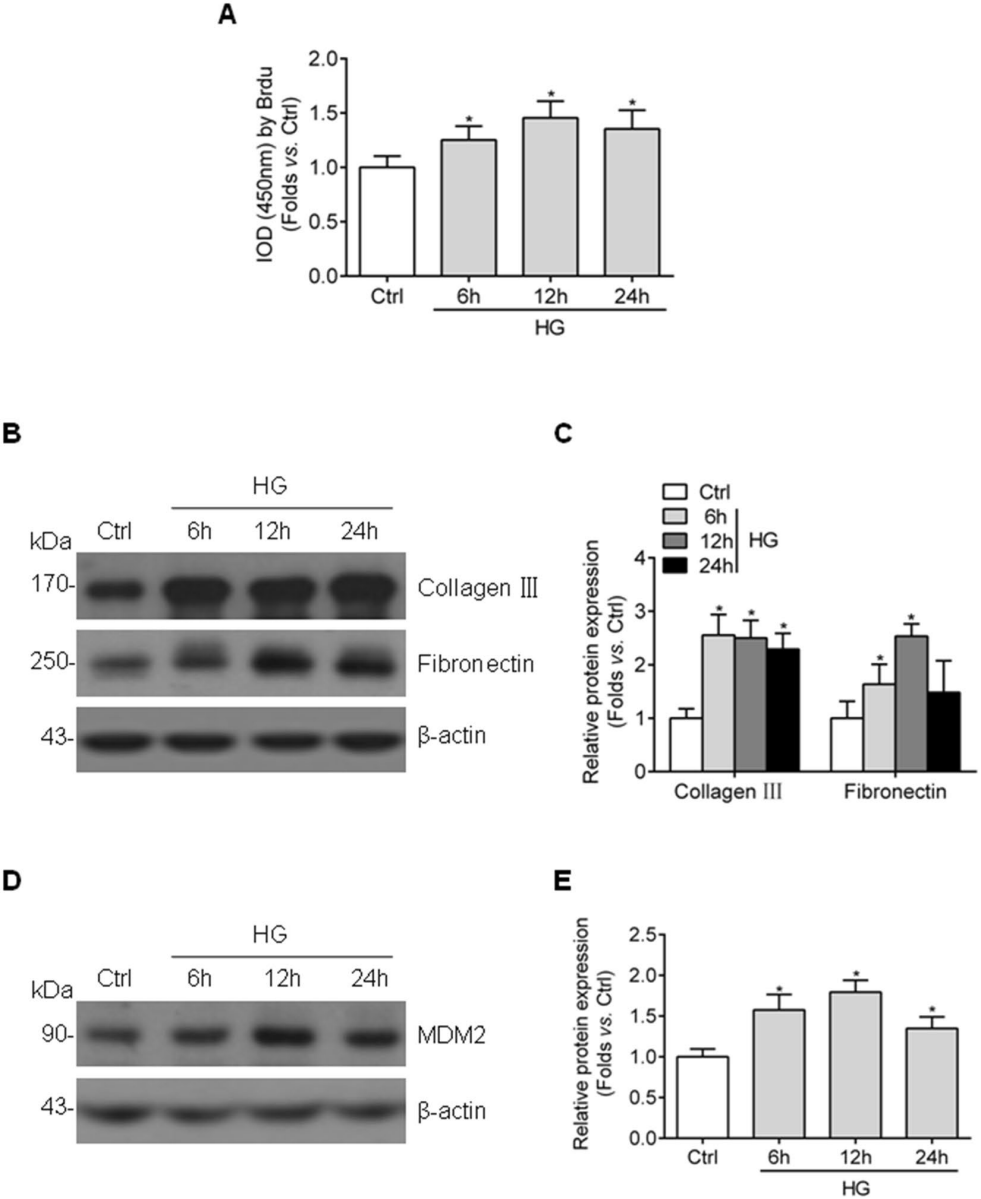
Cell proliferation and collagen III, fibronectin accumulation along with upregulated MDM2 expression in high glucose-cultured GMCs. (**A**) BrdU summarized data showing that high glucose induces HBZY-1 cell proliferation from 6 h to 24 h n = 7. (**B**) Representative western blot images and (**C**) summarized data showing the collagen III and fibronectin protein level in HBZY-1 treated with high glucose for indicated time n ≥ 3. (**D**) Representative western blot images and (**E**) summarized data showing MDM2 protein level in HBZY-1 treated with high glucose for indicated time n = 6. Ctrl: control; HG: high glucose. ^*^ *P* < 0.05 *vs*. Ctrl.

To determine the influence of high glucose on GMC proliferation and ECM protein accumulation, HBZY-1 cells were exposed to high glucose media for indicated times. In line with previously reports^22, 23^, the cell proliferation was significantly induced by high glucose, and the expression of collagen III and fibronectin (indicators for ECM accumulation) also increased (Fig. 1A–C). Next, we detected MDM2 expression in cultured GMCs and we found the protein abundance of MDM2 was upregulated in a similar time-dependent manner (Fig. 1D,E), the influence of osmotic pressure on MDM2 expression was rule out using Mannitol (data not presented). These data suggest that MDM2 may be correlated to GMC proliferation and ECM accumulation under hyperglycemia situation.

### MDM2 contributes to high glucose-induced ECM accumulation and cell proliferation in GMCs

**Figure 2:**
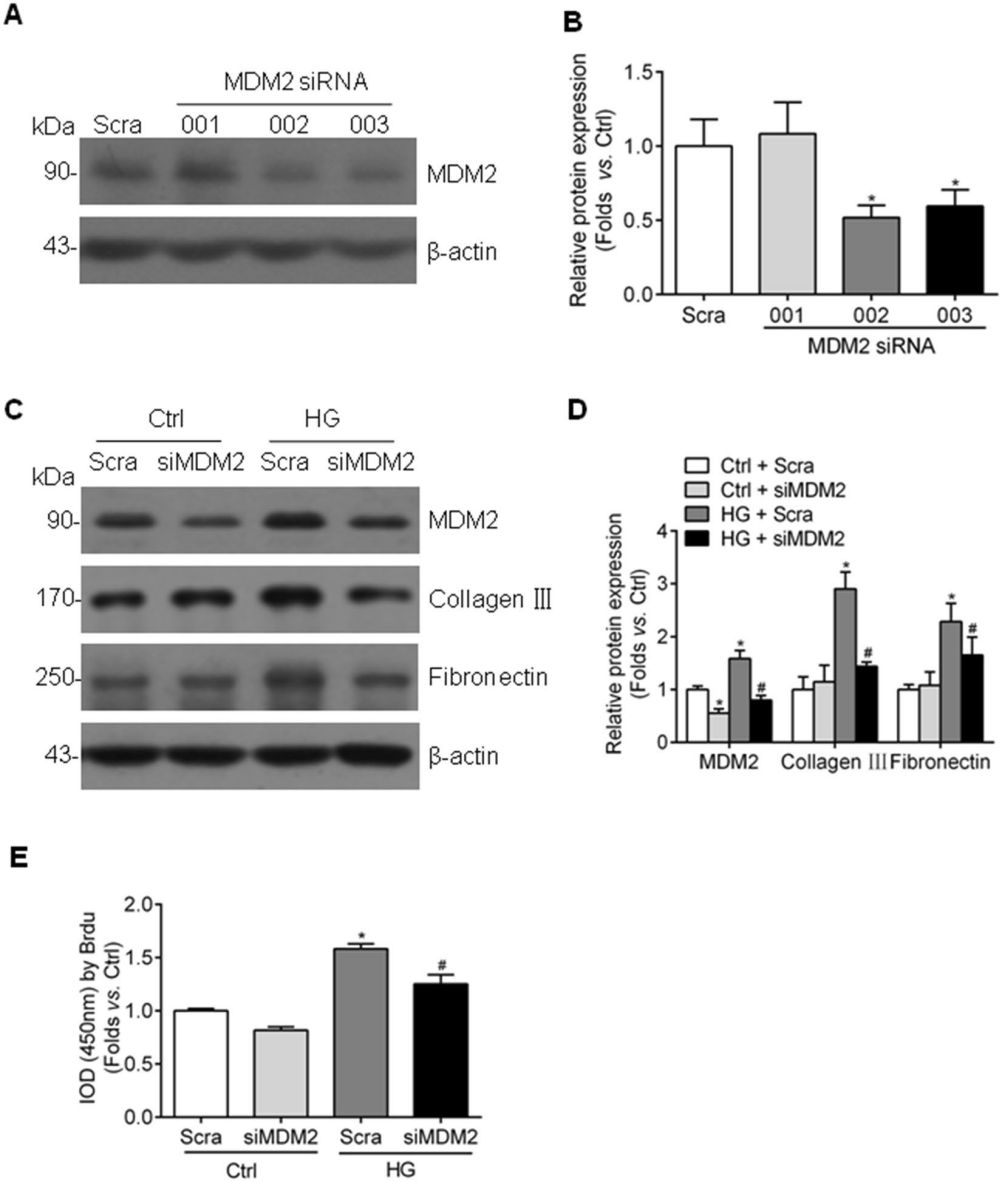
Genetic depletion of MDM2 attenuates high glucose-induced collagen III, fibronectin accumulation and cell proliferation in GMCs. (**A**) Representative western blot images and (**B**) summarized data showing the interfere effect of three different sequence siRNA oligonucleotides targeting MDM2 n = 3. (**C**) Representative western blot images and (**D**) summarized data showing that depletion MDM2 by siRNA partially attenuates collagen III and fibronectin accumulation in high glucose-stimulated HBZY-1 cells n = 6. (**E**) BrdU summarized data showing that high glucose-induced cell proliferation is inhibited by MDM2 genetic depletion n = 6. Scra: scramble shRNA; siMDM2: MDM2 siRNA. ^*^ *P* < 0.05 *vs*. Ctrl + Scra; ^#^ *P* < 0.05 *vs*. HG + Scra.

To evaluate the role of MDM2 in mediating GMC proliferation and ECM accumulation, we designed three different sequence siRNA oligonucleotides targeting MDM2 gene and the most efficient one (002) was utilized to knockdown MDM2 expression in the following experiments. The transfection efficiency of this siRNA was approximately 50% under normal or high glucose condition, and we found MDM2 inhibition dramatically decreased the high glucose-induced upregulation of collagen III and fibronectin (Fig. 2A–D). In addition, we investigated the influence of MDM2 silencing on GMC proliferation. As shown in Fig. 2E, high glucose-induced GMC proliferation was also largely reversed by MDM2 depletion, indicating that MDM2 plays a critical role in GMC dysfunction under hyperglycemia condition.

### Nutlin-3a cannot alleviate renal impairment of diabetic mice and high glucose-induced ECM accumulation *in vivo* and *in vitro*

**Figure 3:**
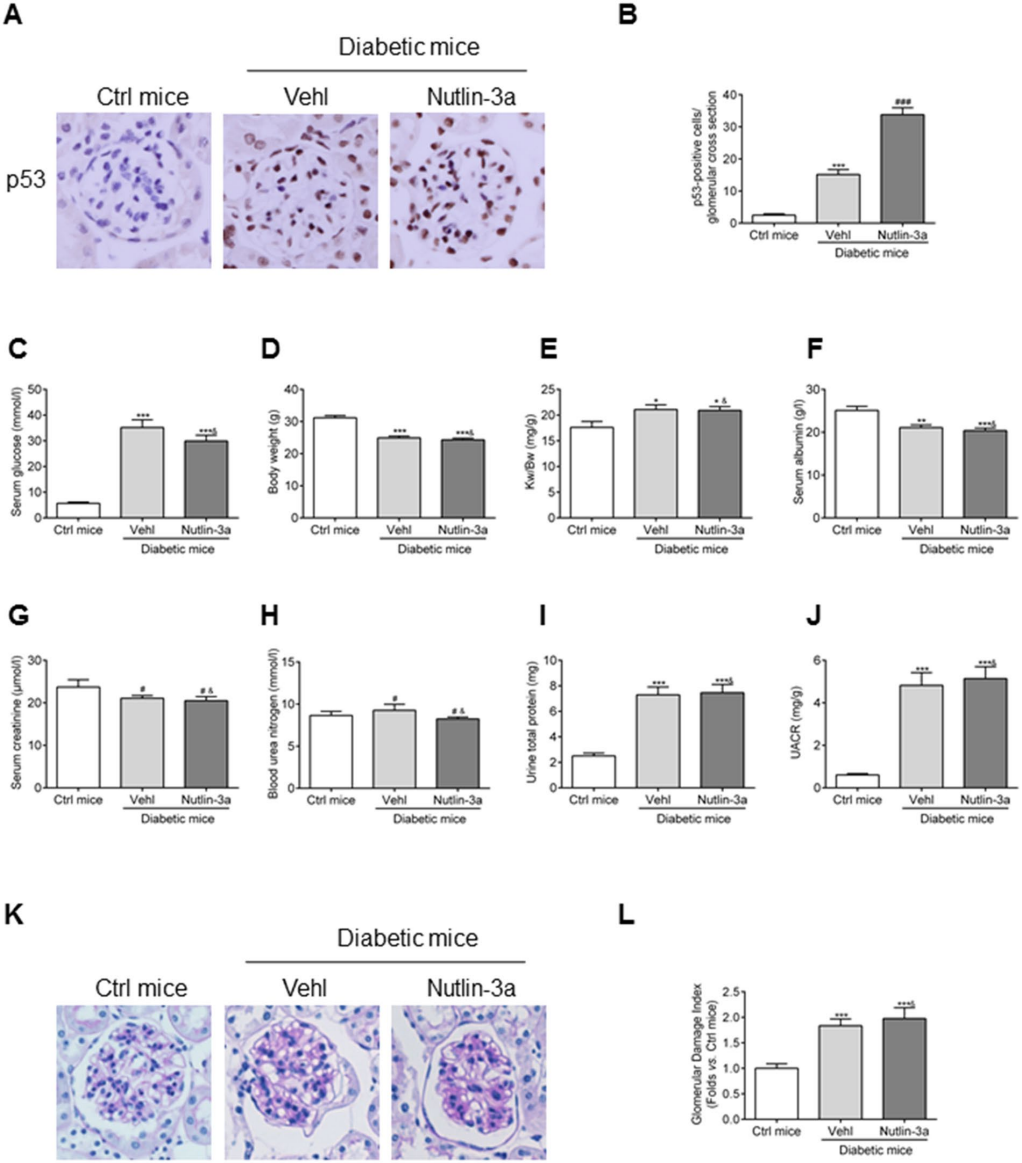
Nutlin-3a cannot ameliorate the renal impairment in STZ-induced diabetic mice. (**A**) Representative photomicrograph of p53 density in the glomeruli of mice in each group (original magnification 200x). (**B**) Quantification of p53-positive cells/glomerular cross section for each group n = 6. (**C–J**) Summarized data showing the serum glucose, body weight, Kw/Bw, serum albumin, serum creatinine, blood urea nitrogen, urine total protein, and UACR of the experimental mice in our study n ≥ 7. (**K**) Representative PAS staining images of glomeruli (original magnification 200x). (L) Summarized data showing the glomerular damage index (GDI) of the three groups of mice. n ≥ 7. Vehl: vehicle; Kw: kidney weight; Bw: bodyweight; UACR: urine albumin to creatinine ratio. ^*^ *P* < 0.05 *vs*. Ctrl mice; ^**^ *P* < 0.01 *vs*. Ctrl mice; ^***^ *P* < 0.0001 *vs*. Ctrl mice; ^#^ *P* > 0.05 *vs*. Ctrl mice; ^###^ *P* > 0.05 *vs*. diabetic mice + Vehl; ^&^ *P* > 0.05 *vs*. diabetic mice + Vehl.

Next, we explored the possible mechanisms involved in MDM2-associated GMC dysfunction. p53 is a classic downstream target of MDM2 and Nutlin-3a is a cis-imidazoline compound that inhibits MDM2-mediated p53 degradation, which has been reported to play a beneficial role in variety of kidney diseases^17, 21, 24^. Thus we treated the STZ-induced diabetic mice with Nutlin-3a to investigate the p53-dependent role of MDM2. First, by immunohistochemistry we found the intensity of p53 was markedly increased in the glomeruli of mice with Nutlin-3a treatment (Fig. 3A,B), indicating the efficient blocking of MDM2-p53 signaling. However, there was no significant difference between vehicle and Nutlin-3a treated diabetic mice in the physical and biochemical parameters including serum glucose, body weight, kidney weight/body weight, serum albumin, serum creatinine, blood urea nitrogen, urine total protein and urine albumin to creatinine ratio (Fig. 3C–J). Meanwhile, no alleviation of pathological changes in glomeruli was observed in Nutlin-3a treated mice (Fig. 3K,L). Collectively, these findings suggest a p53-independent effect of MDM2 in glomerular impairment under diabetic status *in vivo*.

**Figure 4:**
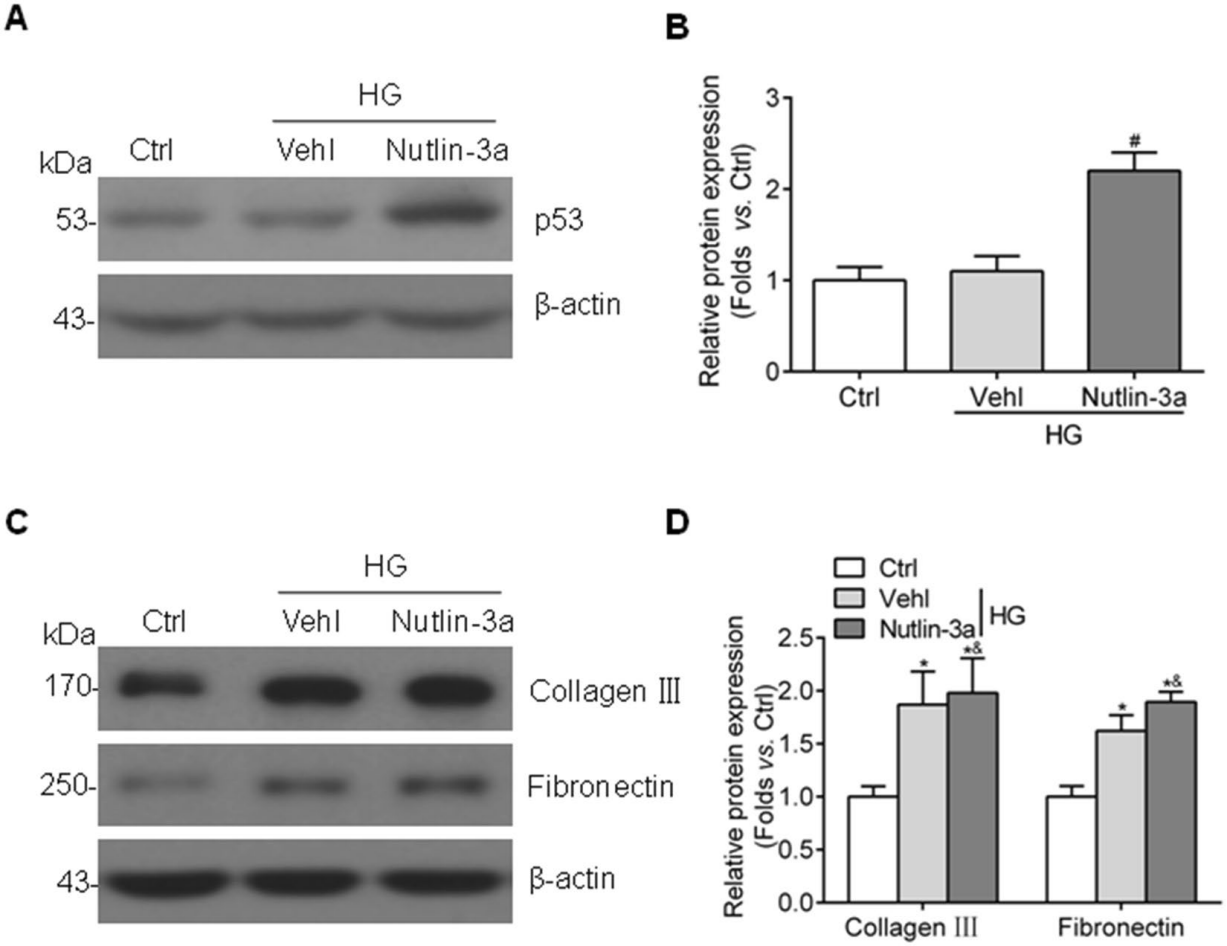
Nutlin-3a cannot reverse high glucose-induced collagen III and fibronectin accumulation *in vitro*. (**A**) Representative western blot images and (**B**) summarized data showing that the elevation expression of p53 in Nutlin-3a-treated HBZY-1 cells n = 4. ^#^ *P* < 0.05 *vs*. HG + Vehl. (**C**) Representative western blot images and (**D**) summarized data showing that Nutlin-3a treatment has no benefit on high glucose-induced collagen III and fibronectin accumulation in cultured HBZY-1 cells n = 7. ^*^ *P* < 0.05 *vs*. Ctrl; ^&^ *P* > 0.05 *vs*. HG + Vehl.

To confirm the role of Nutlin-3a in ECM accumulation *in vitro*, HBZY-1 cells were treated with Nutlin-3a before exposed to high glucose media. Nutlin-3a treatment markedly upregulated the expression of p53 in cultured GMCs (Fig. 4A,B). However, as shown in Fig. 4C,D, Nutlin-3a treatment couldn’t prevent the upregulation of collagen III and fibronectin induced by high glucose in HBZY-1 cells. According to these data, there must be other ways independent of p53 to explain MDM2-mediated GMC dysfunction under hyperglycemia condition.

### Aberrant activation of Notch1 signaling pathway induced by high glucose in GMCs is largely attenuated by MDM2 depletion

**Figure 5:**
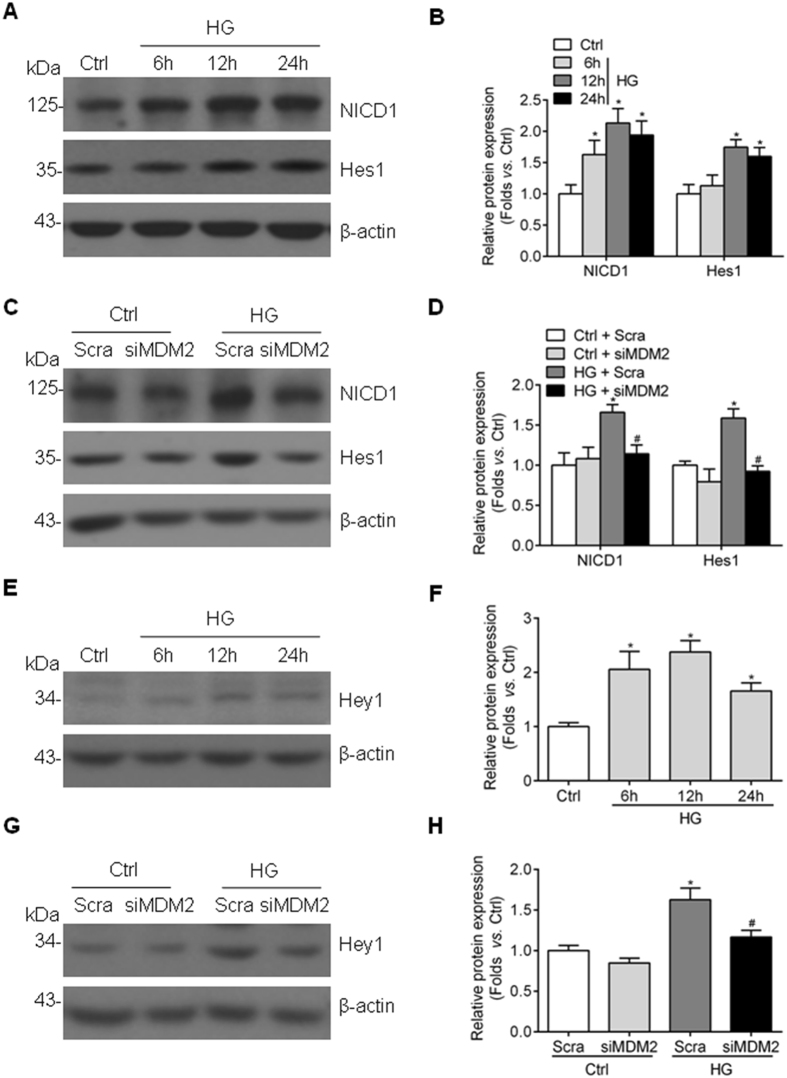
Genetic deletion of MDM2 attenuates aberrant Notch1 signaling in high glucose-treated GMCs. (**A**) Representative western blot images and (**B**) summarized data showing the protein level of NICD1 and Hes1 in HBZY-1 cells exposed to high glucose for indicated times n ≥ 5. ^*^ *P* < 0.05 *vs*. Ctrl. (**C**) Representative western blot images and (**D**) summarized data showing that the genetic deletion of MDM2 by siRNA effectively suppresses the protein level of NICD1 and Hes1 n = 5. ^*^ *P* < 0.05 *vs*. Ctrl + Scra; ^#^ *P* < 0.05 *vs*. HG + Scra. (**E**) Representative western blot images and (**F**) summarized data showing the protein level of Hey1 in HBZY-1 cells exposed to high glucose for indicated times n = 6. ^*^ *P* < 0.05 *vs*. Ctrl. (**G**) Representative western blot images and (**H**) summarized data showing that the genetic deletion of MDM2 by siRNA effectively suppresses the protein level of Hey1 n = 3. ^*^ *P* < 0.05 *vs*. Ctrl + Scra; ^#^ *P* < 0.05 *vs*. HG + Scra.

Notch1 signaling pathway activation has been reported in glomeruli of diabetic kidney and high glucose-stimulated GMCs^25^, which is well-recognized playing a pathogenic part in GMC proliferation and ECM accumulation. In our study, we confirmed Notch1 signaling is activated in HBZY-1 cells exposed to high glucose, characterized by upregulated Notch1 intracellular domain (NICD1) and its downstream target gene Hes1 and Hey1 (Fig. 5A,B,E,F). MDM2 has been reported to activate Notch1 signaling pathway during tumorigenesis^26^, thus we wondered whether MDM2 promotes GMC dysfunction via Notch1 signaling activation in GMCs with high glucose treatment. By knocking down MDM2, the high glucose-enhanced expression of NICD1, Hes1 and Hey1 were significantly reduced (Fig. 5C,D,G,H), indicating that the high glucose-induced activation of Notch1 signaling pathway is attenuated by MDM2 depletion.

### Numb expression is upregulated and cannot be affected by MDM2 inhibition in GMCs exposed to high glucose

**Figure 6:**
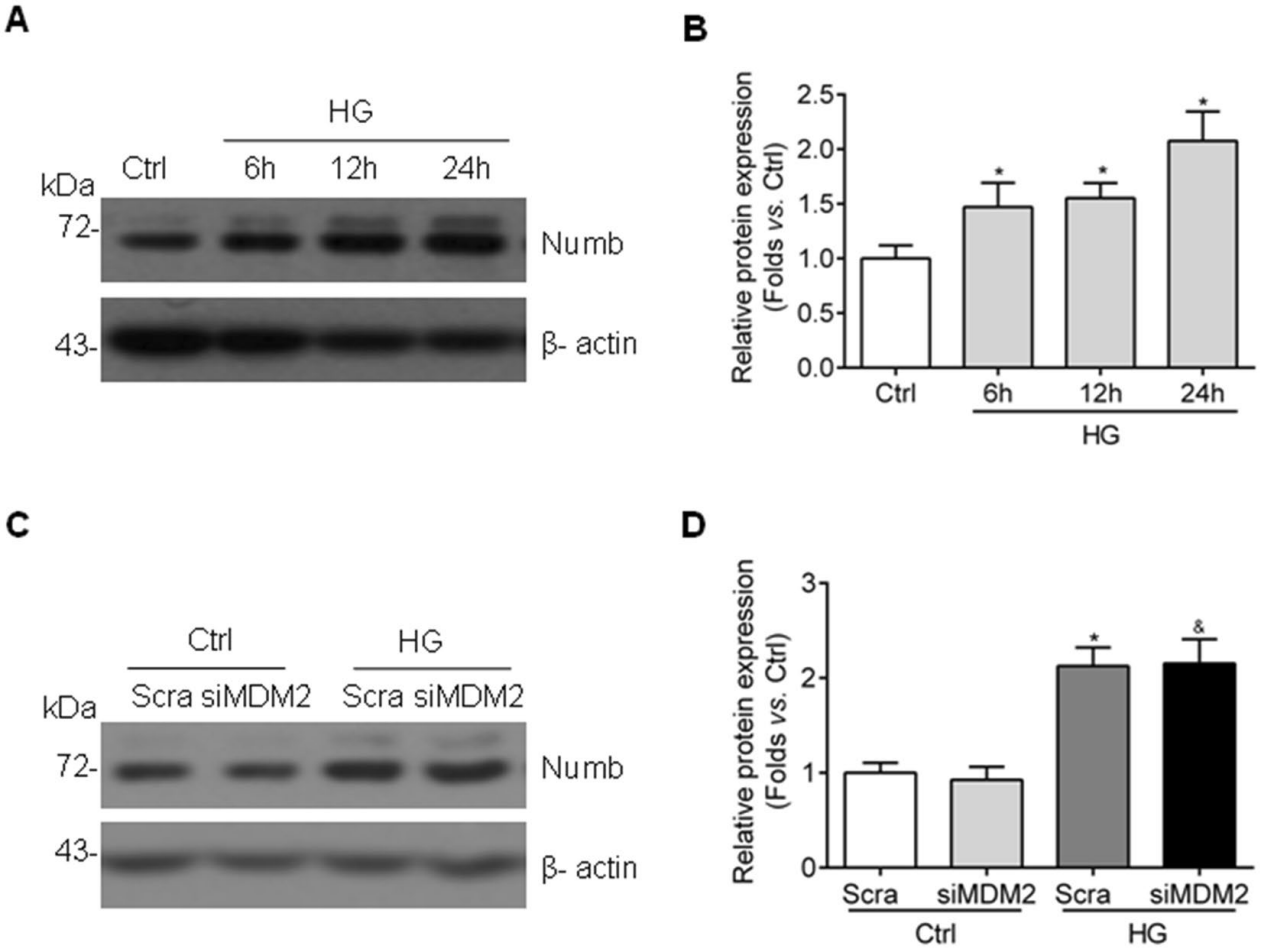
Numb expression is upregulated by high glucose simulation in GMCs and not affected by MDM2 inhibition. (**A**) Representative western blot images and (**B**) summarized data showing the Numb protein level in HBZY-1 with high glucose treatment at indicated time points n = 6. ^*^ *P* < 0.05 *vs*. Ctrl. (**C**) Representative western blot images and (**D**) summarized data showing that the abundance of Numb is not influenced by MDM2 inhibition n = 6. ^*^ *P* < 0.05 *vs*. Ctrl + Scra; ^&^ *P* > 0.05 *vs*. HG + Scra.

We further moved on to investigate how MDM2 regulates Notch1 signaling pathway in high glucose-cultured GMCs. Numb is another important ubiquitination target of MDM2^27^ and also a negative regulator of Notch1 signaling pathway^28^. MDM2 indirectly activates Notch1 signaling pathway by disrupting Numb-Notch1 complex. However, the expression of Numb and whether Numb is a mediator of MDM2-Notch1 interaction in GMCs with high glucose exposure are not known. In our study, the protein abundance of Numb was increased in high glucose-cultured HBZY-1 cells in a time-dependent manner (Fig. 6A,B), and the depletion of MDM2 had no significant influence on the expression of Numb (Fig. 6C,D), suggesting that Numb may not be involved in the association between MDM2 and Notch1 during GMCs dysfunction induced by high glucose.

### MDM2 regulates the ubiquitination status of NICD1

**Figure 7:**
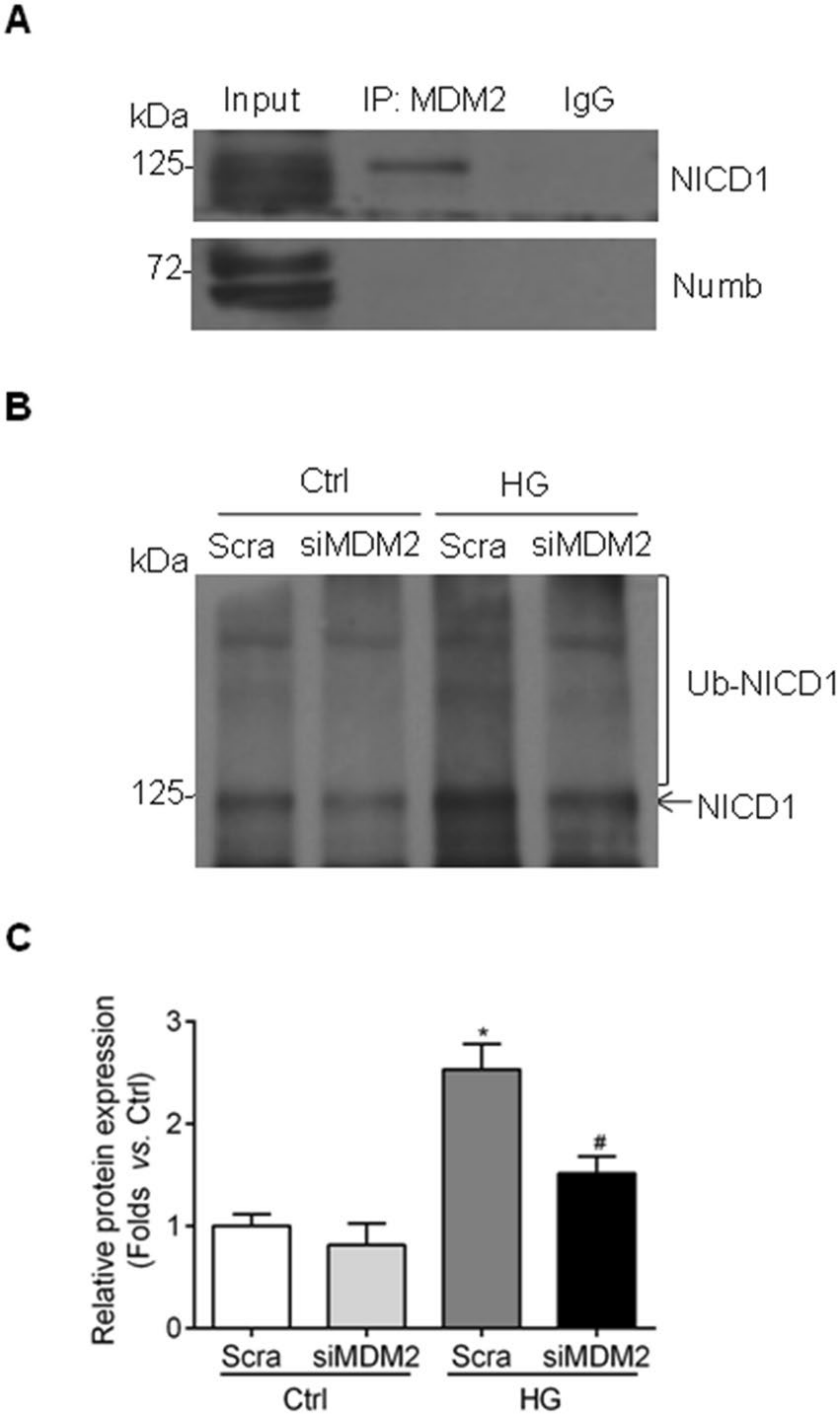
MDM2 binds with NICD1 in GMCs exposed to high glucose and regulates the ubiquination status of NICD1. (**A**) Cell lysates of high glucose-cultured HBZY-1cells were incubated with MDM2 primary antibody, and NICD1 was pulled down by MDM2 primary antibody without Numb binding. Input: whole lysate; IP: immunoprecipitation; IgG: mouse IgG. (**B**) Cell lysates of HBZY-1 cells transfected with MDM2 siRNA were incubated with Notch1 primary antibody, and ubiquitination was detected by immunoblotting with anti-ubiquitin antibody. Ub-NICD1 represents ubiquitinated NICD1. (**C**) Summarized data showing that the ubiquitination NICD1 is regulated by MDM2 inhibition n = 4. ^*^ *P* < 0.05 *vs*. Ctrl + Scra; ^&^ *P* < 0.05 *vs*. HG + Scra.

As MDM2 modulates the activation of Notch1 signaling pathway while Numb is not implicated, we explored other possible mechanisms. It has been reported that MDM2 also modulates Notch1 signaling pathway via direct ubiquitination modification of NICD1, leading to activation of Notch1 signaling pathway instead of degradation^26^, but whether similar regulation contributes to the pathogenesis of DKD is not clarified. In our study, by using immunoprecipitation method, we found that NICD1 could be pulled down by MDM2 primary antibody in high glucose-cultured GMCs, without Numb binding (Fig. 7A). More importantly, the ubiquitination status of NICD1 was regulated by the level of MDM2 when knocking down MDM2 by siRNA (Fig. 7B,C), suggesting an ubiquitination modification role of MDM2 in GMCs under hyperglycemia condition.

## Discussion

In the current study, we explored the role and mechanism of MDM2 in GMC proliferation and ECM accumulation under hyperglycemia condition. Here, we showed that MDM2 is upregulated in high glucose-cultured GMCs, and genetic ablation of MDM2 attenuates cell proliferation and ECM accumulation induced by high glucose. However, neither p53 nor Numb is involved in these processes mediated by MDM2 aforementioned. Instead, our findings suggest that MDM2-mediated Notch1 activation is one of the potential mechanisms responsible for high glucose-induced GMC proliferation and ECM accumulation.

MDM2 is a well-known onco-protein that suppresses p53-dependent cell cycle arrest, which also exerts numerous p53-independent functions by interacting with other substrates such as Numb, NF-κB, and so on. Accumulating studies have documented a strong correlation between MDM2 and physiologic homeostasis of kidney^18, 19^ as well as pathological events during diseased status^17, 24^. MDM2 is proposed to be a survival factor for resting podocytes, and also essential for the lifespan and the maintenance of renal function in mice^18, 19^. Meanwhile, the same research group identified MDM2 as a contributor to podocyte mitotic catastrophe in adriamycin nephropathy^21^. Moreover, MDM2 is involved in anti-GBM serum induced-crescentic glomerulonephritis by triggering the aberrant cell cycle entry of PECs and inflammatory cell infiltration^17^. However, whether MDM2 is implicated in modulating GMC function during hyperglycemia is largely unknown. Our data first revealed that high glucose upregulated the protein level of MDM2 in a time-dependent manner, accompanied with an elevation of collagen III and fibronectin accumulation, as well as cell proliferation *in vitro*. These data drive us to explore whether MDM2 is a mediator of high glucose-induced GMC dysfunction. Indeed, inhibiting MDM2 by genetic strategy confirmed the pivotal role of MDM2 in mediating high glucose-induced cell proliferation and ECM accumulation in GMCs, which are two crucial events leading to glomerulosclerosis in DKD.

Then we explored the underlying mechanisms. It has been reported that inhibition of MDM2-p53 interaction using Nutlin-3a attenuates kidney disorders in various diseased situations. For example, Nutlin-3a treatment prevents adriamycin-induced podocyte detachment and alleviates glomerular pathological change^21^. Moreover, Nutlin-3a treatment ameliorates kidney injury in MRL-Fas^lpr^ mice^24^ and abrogates anti-GBM serum induced crescentic glomerulonephritis^17^. Thus we examined the intervention effect of Nutlin-3a in DKD animal model. Unexpectedly, Nutlin-3a treatment failed to improve biochemical parameters of STZ-induced diabetic mice. Also, there was no significant alleviation of pathological changes in the glomeruli of these mice, especially mesangial lesions. Corresponding to these *in vivo* data, Nutlin-3a couldn’t alleviate collagen III and fibronectin accumulation in cultured GMCs exposed to high glucose *in vitro*. It is worth mentioning that, in a recent published report, inhibition of MDM2 with Nutlin-3a in normal C57BL/6 mice leads to damage of glomerular filtration barrier and renal function, meanwhile a 4-week Nutlin-3a treatment also shows no renal protection effect in db/db diabetic mice^29^. Thus, we concluded that the benefit of MDM2 ablation described above is obtained via a p53-independent way.

Notch1 is a single trans-membrane protein that contributes to the formation and development of kidney, and expresses less in adult kidney^30, 31^. The release of active NICD1 by γ-secretase is critical for the subsequent transcriptional activation of downstream Notch1-targeted genes, such as Hes1/Hey1^32, 33^. Abnormal activation of Notch1 signaling pathway is implicated in diabetic-related podocyte injury and GMC dysfunction^25, 34, 35^, while podocyte-specific deletion of Notch1 protects mice from diabetic-associated podocyte damage and depletion^34, 35^. In accordance with these findings, inhibition of Notch1 pathway by blocking the release of NICD1 using γ-secretase inhibitor also alleviates albuminuria and glomerulosclerosis in rat DKD model^36^. Thus, getting a better understanding of Notch1 signaling regulation during hyperglycemia is of great importance. The connection between MDM2 and Notch signaling pathway including Notch1 and Notch4 has been observed during tumorigenesis^26, 37, 38^. But it is rarely known if there is a link between MDM2 and Notch1 in high glucose-induced GMC dysfunction. In this regard, we first confirmed the activation of Notch1 signaling pathway in high glucose-cultured GMCs, and further revealed that this process is largely modulated by MDM2, if not all. Our data indicated that Notch1 plays an important role in the pathogenetic process associated with MDM2 in mesangial cells, especially under high glucose conditions.

To further clarify the mechanism of Notch1 activation, we tested the expression of Numb, a protein that suppresses Notch1 signaling pathway by binding with NICD1, preventing its nuclear localization and recruiting ubiquitination, and leading to degradation of NICD1^28, 39^. It is widely accepted that MDM2 promotes aberrant activation of Notch1 signaling via ubiquitinating and degrading of Numb^27, 40, 41^. However, the expression change of Numb and its potential role in mediating the effect of MDM2 in high glucose-cultured GMCs is unclear. In the present study, we found that high glucose induced the expression of Numb protein in a time-dependent manner, while knockdown of MDM2 had no effect on Numb expression. Therefore, Numb may not be involved in MDM2-regulated Notch1 activation, at least in GMCs. MDM2 is suggested to modulate Notch1 signaling pathway through other mechanisms.

Ubiquitination is one type of posttranslational modifications, which is important for regulating protein activity and stability. Usually, protein ubiquitination is considered as a proteasome-dependent degradation fate of protein. However, it is also reported that protein ubiquitination plays a role in the activation of signal transduction pathways, such as Akt hyperactivation^42^ in cancer and activation of IKK (IκB kinase) in NF-κB pathway^43^. MDM2 has been reported to directly interact with NICD1, which leads to the ubiquitination and the transcriptional activation of Notch1 signaling pathway in breast cancer cells^26^. In our study, it was found that MDM2 interacted with NICD1 without Numb binding. More importantly, the ubiquitination status of NICD1 was regulated by MDM2, which parallels with the activation status of Notch1 signaling pathway described above. Thus, ubiquitination modification-related Notch1 activation may be a reasonable explanation for the relationship between MDM2 and Notch1 signaling pathway in GMCs exposed to high glucose.

In summary, we demonstrated that MDM2 is essential in mediating high glucose-induced GMC proliferation and ECM accumulation, through the activation of Notch1 via ubiquitination modification of NICD1. Our current study reveals a novel mechanism involved in GMCs dysfunction under hyperglycemia conditions and might provide a potential target for the early intervention of DKD.

## Research Materials and Methods

### Animals

The animal experiments were performed according to the guidelines for use and care of laboratory animals of National Institutes of Health, and approved by the Animal Care and Use Committee of Tongji Medical College. 7–8 weeks old C57BL/6 mice weighing 21–24 g were obtained from Charles River (Beijing, China), and raised in a pathogen-free environment with a 12-hour light and dark cycle, and unrestricted access to food and water. To create the diabetic model, the mice were starved for 12 h and intraperitoneally injected with a single dose of streptozotocin (STZ, 150 mg/kg body weight, BOSTER, Wuhan, China), while control mice received only citrate buffer. Blood glucose was monitored weekly by glucometer readings. Only the mice with stable serum glucose levels over 16.7 mmol/l were included in the following experiment. The diabetic mice were divided into two groups: one group treated with 60% DMSO (vehicle) and the other with Nutlin-3a (20 mg/kg body weight, Biochempartner, Shanghai, China) every day for 12 weeks. Then urine samples, blood samples and kidney tissues were collected.

### Serum, urine samples detection

The blood harvested from the mice were clotted for 2 h at room temperature, and then the blood and urine samples were centrifuged for 20 min at 1000 rpm to get the supernatants. The samples were aliquoted and stored in −80 °C until detected. The serum glucose, albumin, creatinine, blood urea nitrogen, urine total protein and urine creatinine were measured by Auto-Chemistry Analyzer. Urine albumin was detected by Elisa kit (Elabscience, Wuhan, China) according to the manufacturer’s instructions.

### Immunohistochemistry

Kidney tissues were fixed in 4% neutral buffered formalin, dehydrated in graded sucrose, and embedded in paraffin. Immunohistochemical staining of p53 was performed using a standard biotin-streptavidin-peroxidase method. Briefly, 4 μm thick sections were deparaffinized and rehydrated. Endogenous peroxidase was blocked by 5% BSA for 30 min at room temperature, then the sections were incubated with the primary antibody for p53 (rabbit, 1:100; Protein Tech Group, Chicago, IL, USA) overnight at 4 °C. Then, sections were washed in PBS and incubated with biotinylated goat anti-rabbit antibody and HRP-labeled streptavidin (Beyotime, Jiangsu, China), each for 20 min. At last, peroxidase activity was visualized by diaminobenzidine, and sections were counterstained with hematoxylin and observed under light microscope and 20 glomeruli were randomly chosen for counting the p53-positive cells as described previously^44^.

### Morphological examination

Periodic acid-schiff (PAS) staining of 4 μm paraffin sections was used to examine the morphometric change in the glomeruli. 50 randomly chosen glomeruli of each kidney section were calculated to get the score of glomerular damage index (GDI) as described previously^45^.

### Cell culture, treatment, RNA interference

A rat GMCs line (HBZY-1) was routinely cultured in MEM/EBSS media containing 10% fetal bovine serum, 100 U/ml penicillin and 100 U/ml streptomycin in 37 °C incubator with 5% CO_2_. The cells were exposed to media with 30 mmol/l final glucose concentration for indicated time.

For chemical intervention, the cells were pretreated with 5 μmol/l Nutlin-3a for 1 h, and then exposed to high glucose media for 12 h before harvested.

For RNA interference, short-interfering RNA (siRNA) target on rat MDM2 was obtained from Ribobio (Guangzhou, China). Cells reaching an appropriate confluence were transfected with MDM2 siRNA for 48 h and the scramble siRNA was used as a negative control. The transfection procedure was carried out according to the manufacturer’s instructions. Cells were exposed to high glucose media for 12 h before harvested.

### Cell proliferation

BrdU assay was used to measure cell proliferation. The BrdU incorporation was carried out according to the manufacturer’s instructions of commercial kit (Abcam, Cambridge, MA, USA). Briefly, HBZY-1 cells were seeded into 96-weel plate at a proper density, after indicated treatment, BrdU solution was added into culture media and cultured for another 2 h in the incubator, and then cells were fixed. BrdU incorporation was detected by BrdU detector antibody and peroxidase substrate. The absorbance was read at 450 nm.

### Western blotting analysis

The total protein of cultured cells was extracted by RIPA lysis buffer containing protease inhibitors. BCA detection method was used to determine the protein concentration of cell lysates. Then the cell lysates were supplied with SDS protein loading buffer and boiled in 98 °C for 5 minutes, leading to protein denaturation. Western blotting analysis was carried out briefly as follows: the denatured proteins were separated by SDS-PAGE and transferred to PVDF membranes. After blocking step, the membranes were incubated with indicated primary antibodies overnight at 4 °C. These antibodies were used as follows: MDM2 (mouse, 1:400); Hes1 (mouse, 1:1,000); *β*-actin (1:10,000) were obtained from Santa Cruz Biotechnology (Santa Cruz, CA, USA), Collagen III (rabbit, 1:1,000); Fibronectin (rabbit, 1:1,000), p53 (rabbit, 1:1,000), Hey1 (rabbit, 1:1,000), Ubiquitin (rabbit, 1:1,000) were obtained from Protein Tech Group (Chicago, IL, USA), Notch1 (rabbit, 1:500) was obtained from Abcam (Cambridge, MA, USA), Numb (rabbit, 1:1,000) was obtained from Cell Signal Transduction (Danvers, MA, USA). The next day, the membranes were washed three times with Tris-buffered saline with 0.1% Tween 20 (TBST, pH 7.2–7.6) before incubating with corresponding HRP-conjugated secondary antibodies with a 1:20,000 dilution for 1 h at room temperature. The ECL reagents were used to detect immunoreactive bands with film. The relative intensity of target bands were quantified by Image J software and normalized by the intensity of β-actin.

### Immunoprecipitation

The cell lysates were prepared just as described above. Then equal amounts (0.5–1 mg) of total protein samples were supplied with 1 μg primary antibody and 30 μl Protein A/G PLUS-Agarose (Santa Cruz Biotechnology, Santa Cruz, CA, USA), then incubated overnight at 4 °C on a rotating device. The primary antibody used for immunoprecipitation in our present study including MDM2 (mouse, Santa Cruz Biotechnology, Santa Cruz, CA, USA), Notch1 (rabbit, Sigma, St Louis, MO, USA). The following day, the immunoprecipitates were collected by centrifuging at 2,500 rpm for 5 min at 4 °C. The pellets were washed 3 times with 1.0 ml PBS on a rotating device for 5 min each time. After the final wash, the pellets were resuspended in SDS sample buffer, then boiled in 98 °C for 5 min. The immunoprecipitated proteins were detected by western blotting analysis.

### Statistical Analysis

All of the result data in this study were expressed as mean ± SEM. Significant difference were analyzed using two-tailed T test with GraphPad Software. *P* < 0.05 was considered as of statistical significance.

### Data availability

The authors declare the data availability.

## Electronic supplementary material

Supplementary Data
