# Supplementary material for: MDM2 Contributes to High Glucose-Induced Glomerular Mesangial Cell Proliferation and Extracellular Matrix Accumulation via Notch1
Source: Sci Rep. 2017 Sep 4;7:10393. doi: 10.1038/s41598-017-10927-5 (PMC5583188; doi:10.1038/s41598-017-10927-5)

**MDM2 Contributes to High Glucose-Induced Glomerular Mesangial Cell Proliferation and Extracellular Matrix Accumulation via Notch1**

Chun-Tao Lei, Hui Tang, Chen Ye, Chao-Qun You, Jiao Zhang, Chun-Yun Zhang, Wei Xiong, Hua Su, Chun Zhang *

Department of Nephrology, Union Hospital, Tongji Medical College, Huazhong University of Science and Technology, Wuhan 430022, China


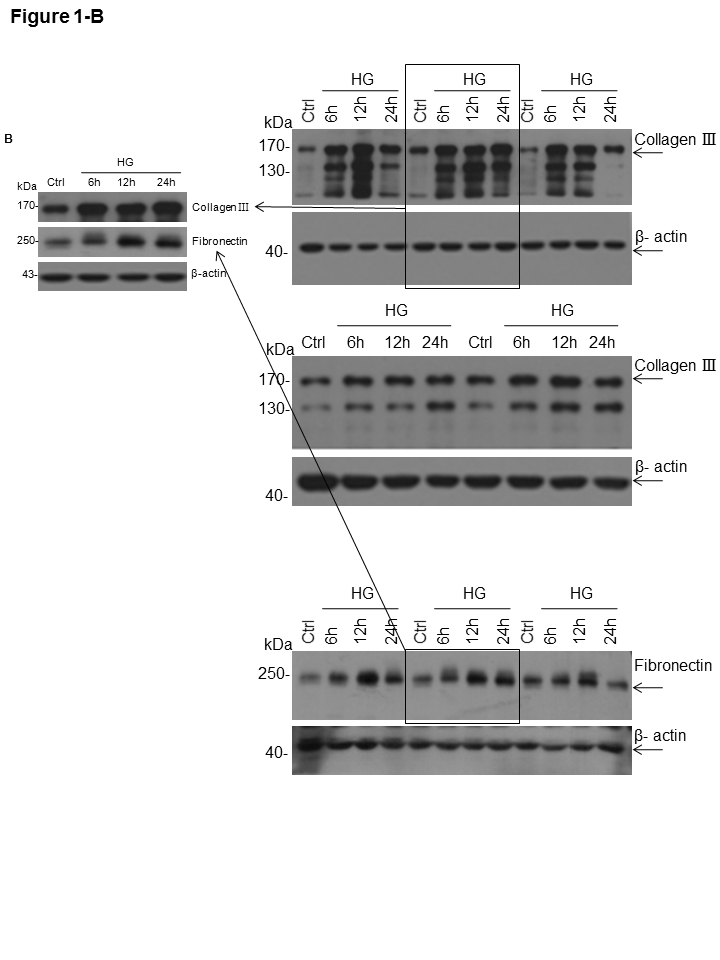

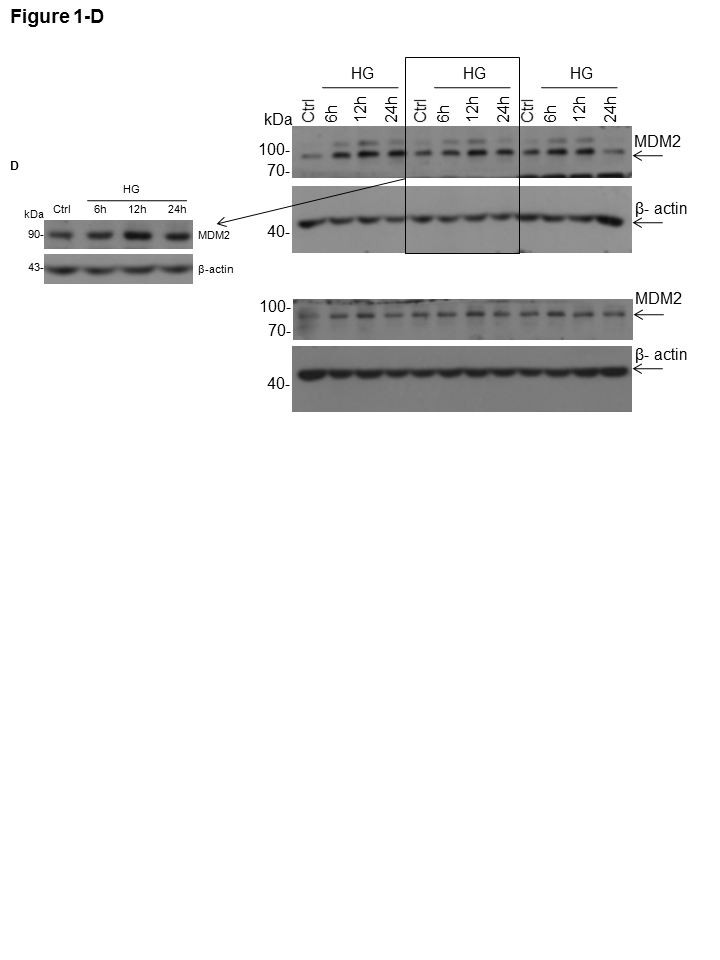

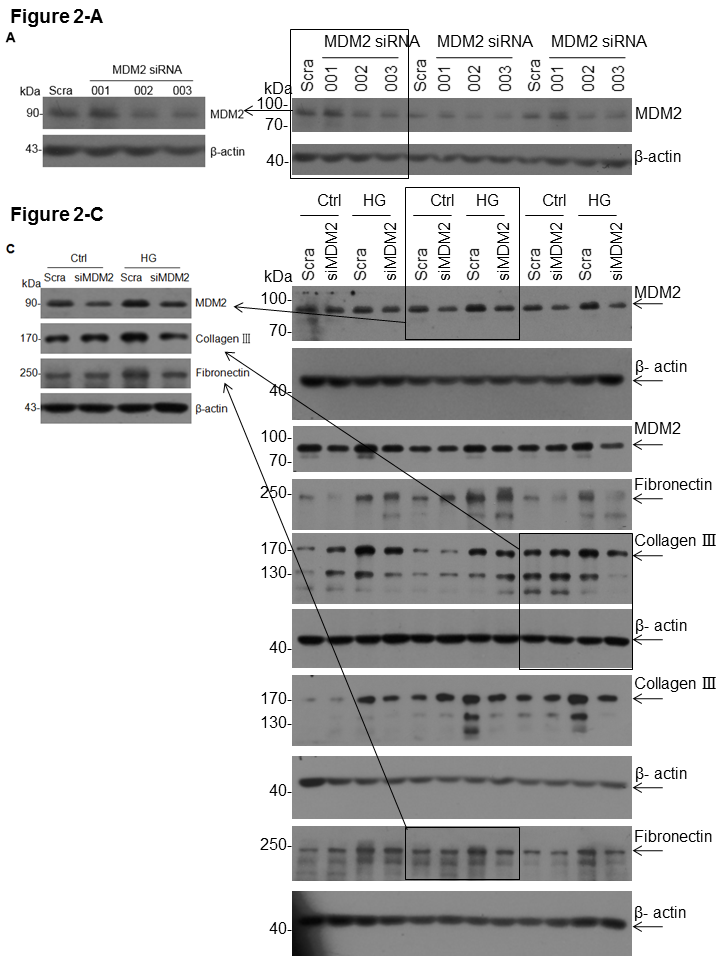

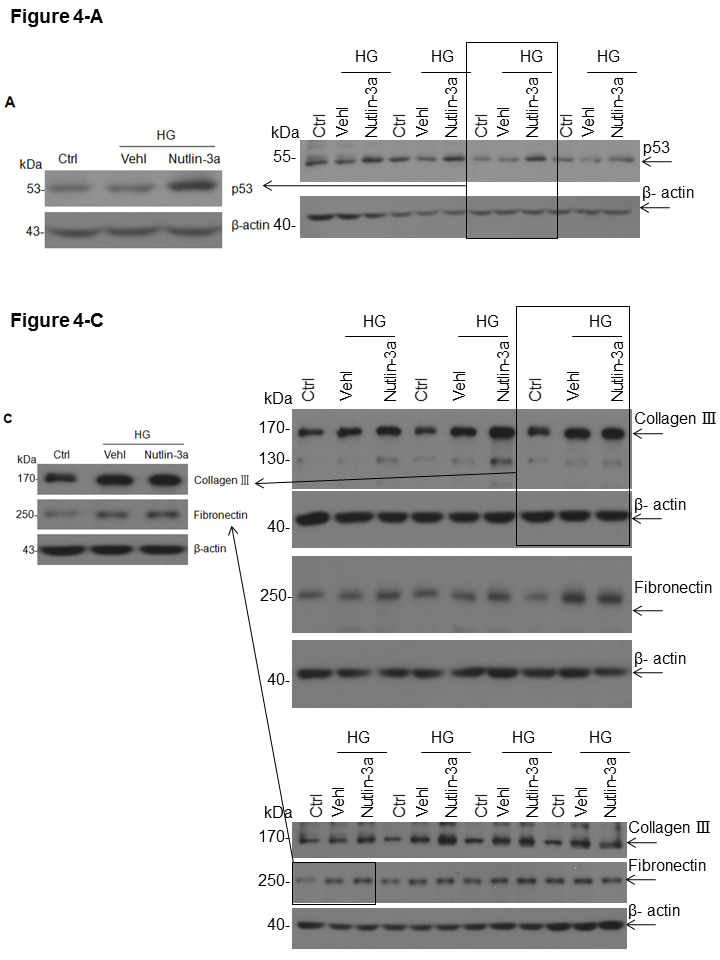

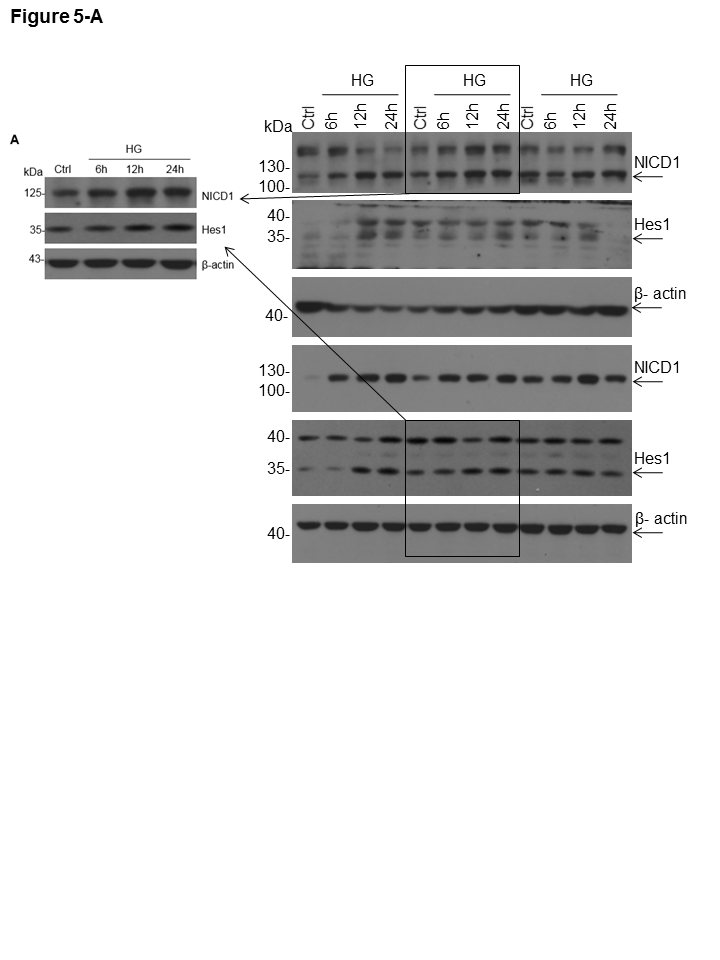

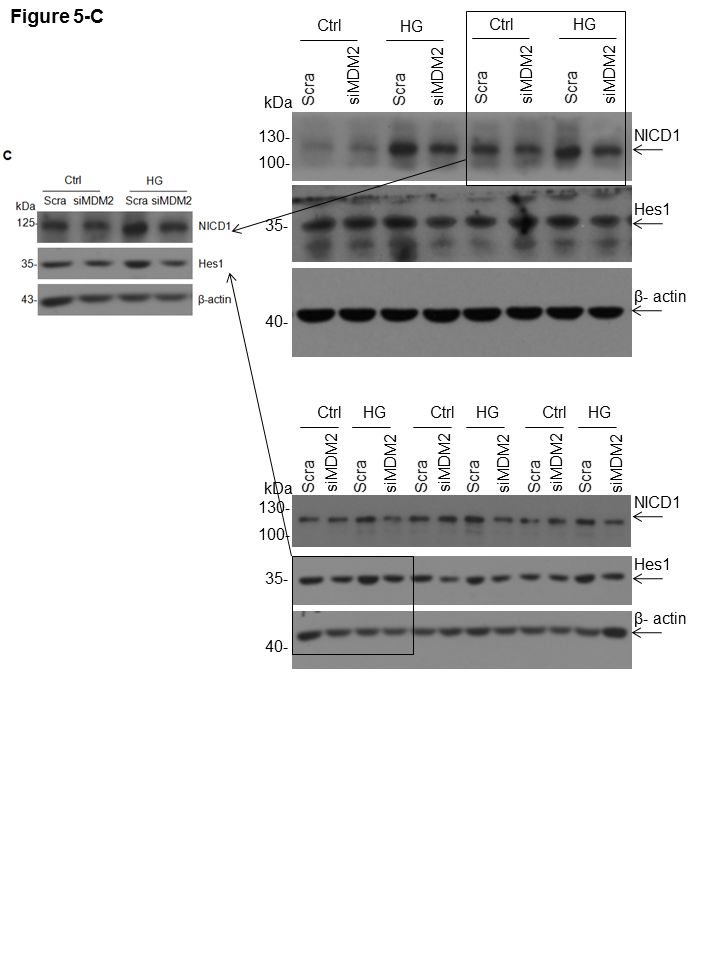

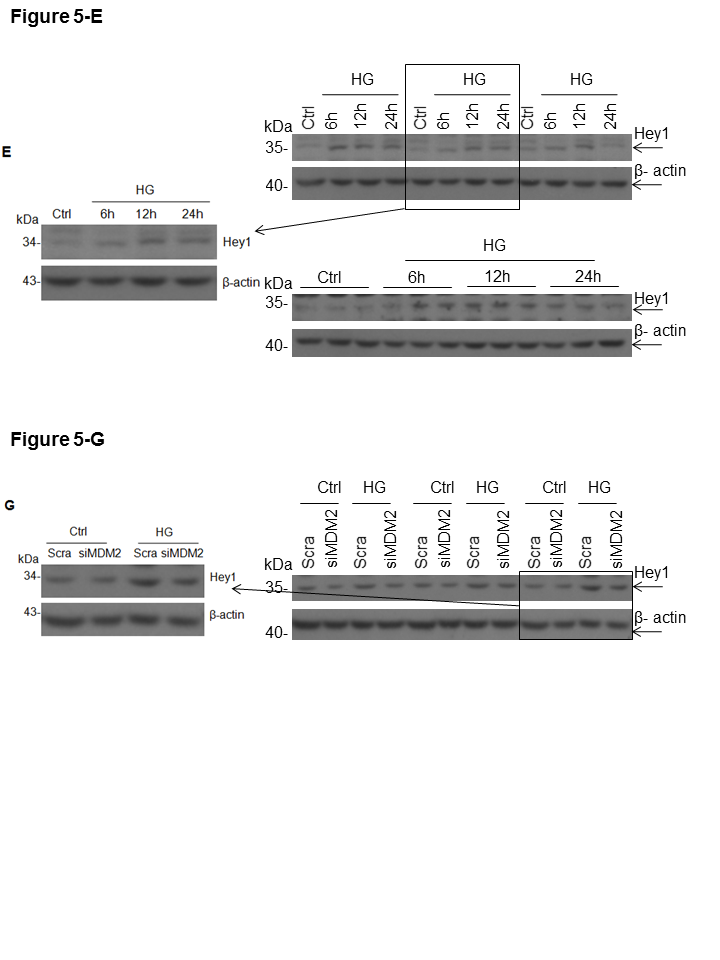

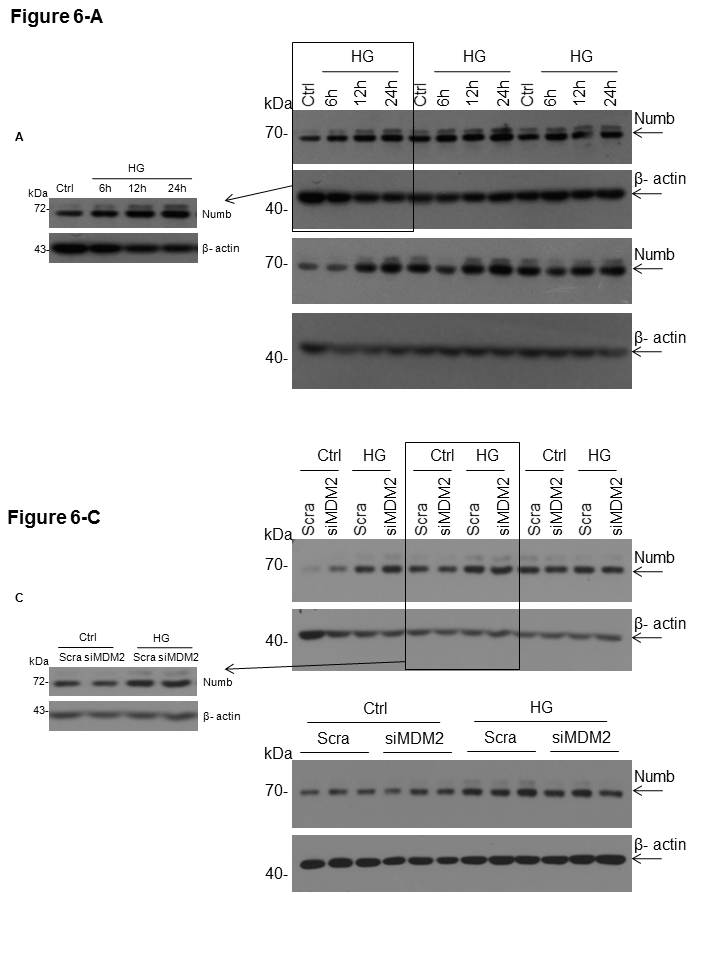

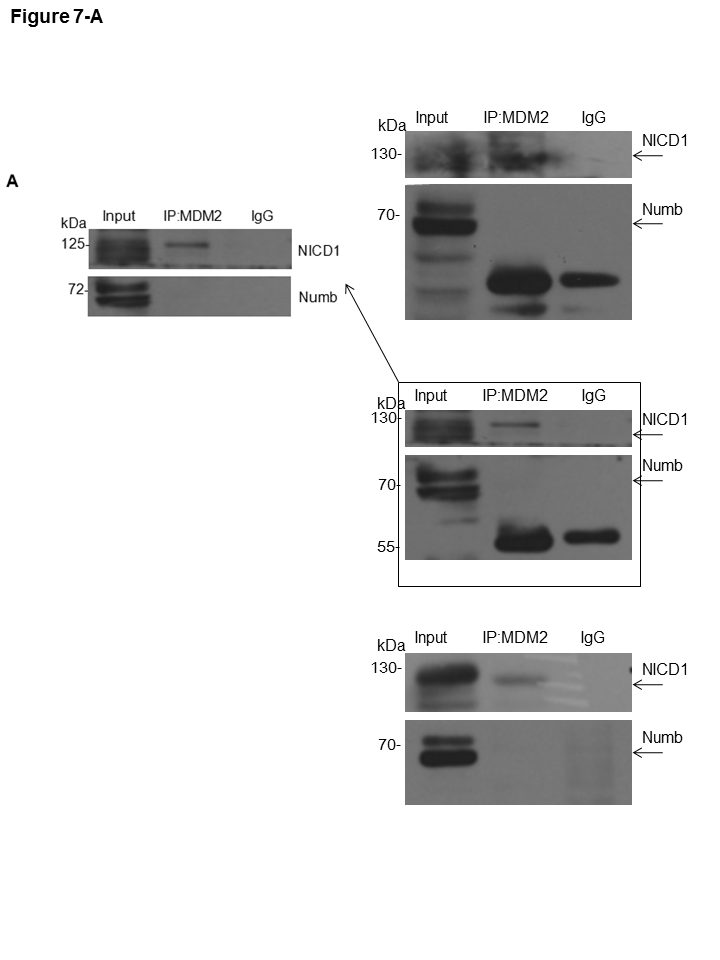

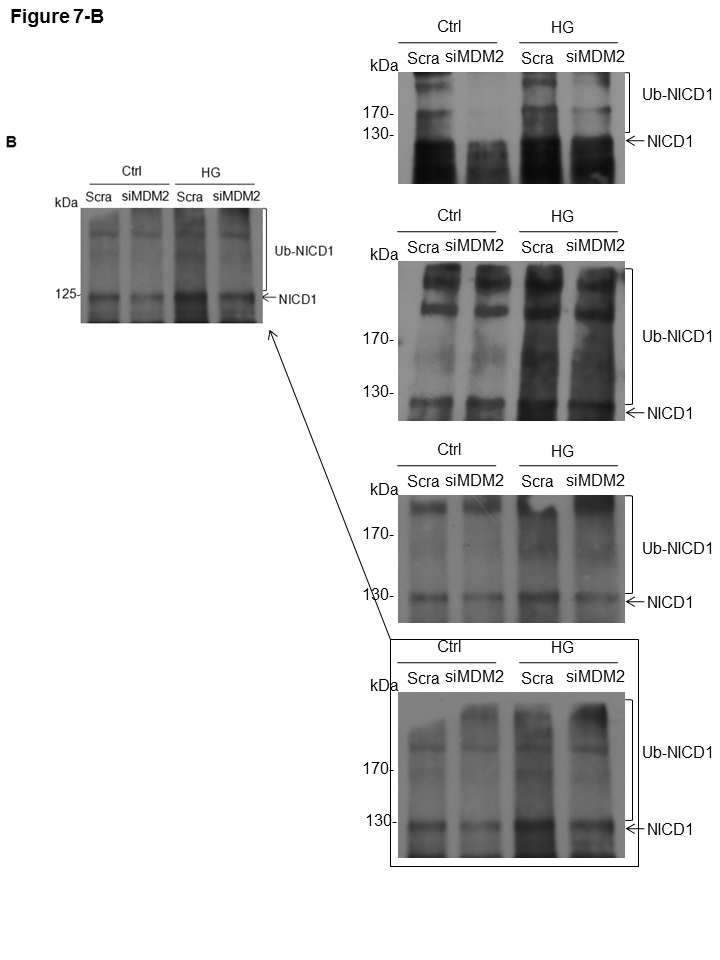

Supplement: Supplementary file 1 — Supplementary Data [file 41598_2017_10927_MOESM1_ESM.doc]
